# Risk environments facing potential users of a supervised injection site in Ottawa, Canada

**DOI:** 10.1186/s12954-015-0083-9

**Published:** 2015-10-22

**Authors:** Ashley Shaw, Lisa Lazarus, Tyler Pantalone, Sean LeBlanc, Dolly Lin, Daina Stanley, Caleb Chepesiuk, Sheetal Patel, Mark Tyndall, The PROUD Community Advisory Committee

**Affiliations:** Ottawa Hospital Research Institute, 725 Parkdale Ave., Ottawa, Ontario K1Y 4E9 Canada; PROUD Committee, 85 Primrose Ave., Ottawa, Ontario K1R 6M1 Canada; Drug Users Advocacy League, 216 Murray St., Ottawa, ON K1N 5N1 Canada; Department of Medicine, University of Ottawa, 451 Smyth Rd., Ottawa, Ontario K1H 8M5 Canada; University of Ottawa at The Ottawa Hospital, Division of Infectious Disease, 501 Smyth Rd., Ottawa, ON K1H 8L6 Canada

**Keywords:** Supervised injection, Harm reduction, Injection drug use, Service users, Marginalized populations, Community-based research

## Abstract

**Background:**

Supervised injection sites (SISs) have been effective in reducing health risks among people who inject drugs (PWID), including those who face issues of homelessness, mental health illness, interactions with local policing practices, and HIV infection. We investigate the risk behaviours and risk environments currently faced by potential users of an SIS in Ottawa to establish the need for such a service and to contribute to the design of an SIS that can address current health risks and reduce harm.

**Methods:**

The PROUD cohort is a community-based participatory research (CBPR) project that examines the HIV risk environment among people who use drugs in Ottawa. From March to October 2013, 593 people who reported using injection drugs or smoking crack cocaine were enrolled through street-based recruitment in the ByWard Market neighbourhood, an area of the city with a high concentration of public drug use and homelessness. Participants completed a demographic, behavioural, and risk environment questionnaire and were offered HIV point-of-care testing. We undertook descriptive and univariate analyses to estimate potential use of an SIS by PWID in Ottawa and to explore risk behaviours and features of the risk environment faced by potential users of the service.

**Results:**

Of those participants who reported injecting drugs in the previous 12 months (*n* = 270), 75.2 % (203) reported a willingness to use an SIS in Ottawa. Among potential SIS users, 24.6 % had recently injected with a used needle, 19.0 % had trouble accessing new needles, 60.6 % were unstably housed, 49.8 % had been redzoned by the police, and 12.8 % were HIV positive. Participants willing to use an SIS more frequently injected in public (OR = 1.98, 95 % CI = 1.06–3.70), required assistance to inject (OR = 1.84, 95 % CI = 1.00–3.38), were hepatitis C positive (OR = 2.13, 95 % CI = 1.16–3.91), had overdosed in the previous year (OR = 2.00, 95 % CI = 1.02–3.92), and identified as LGBTQ (OR = 5.61, 95 % CI = 1.30–24.19).

**Conclusion:**

An SIS in Ottawa would be well-positioned to reach its target group of highly marginalized PWID and reduce drug-related harms. The application of CBPR methods to a large-scale quantitative survey supported the mobilization of communities of PWID to identify and advocate for their own service needs, creating an enabling environment for harm reduction action.

## Background

People who use injection drugs (PWID) in Canada continue to face barriers to accessing primary healthcare services [1, 2]. This lack of access is particularly significant given the high burden of drug-related harm that is affecting many PWID communities, including high rates of fatal overdose, cutaneous abscesses and infections, and blood-borne infections including HIV and hepatitis C virus (HCV) [3]. To address these harms, a range of harm reduction services has been developed and implemented primarily in urban centres across the country, including needle and syringe programmes, opiate substitution services, and community-based primary care clinics. These services aim to create low-barrier, non-judgemental access to evidence-based interventions that improve the health and safety of PWID without requiring any reduction in drug consumption [4]. Though many of these services have had success in improving health outcomes and making frequent contact with PWID, barriers to long-term engagement in care remain [5]. One approach that has successfully improved engagement in care is the integration of supervised injection sites (SISs) into the range of health services available to PWID. SISs can serve as a first, low-barrier point of access to the wider range of health and harm reduction services for highly marginalized PWID [6, 7].

SISs are highly targeted harm reduction services that are effective in reaching the most marginalized communities of people who use drugs, including high-intensity injection drug users and those who face issues of homelessness, mental health illness, involvement with sex work, interactions with local policing practices, and concomitant health conditions including HIV and HCV infection [8]. An SIS is a controlled healthcare setting where PWID can access clean injection equipment and inject pre-obtained drugs under the supervision of a healthcare professional. SISs are also referred to as supervised injection facilities, safer injection sites, and medically supervised injecting centres, among other labels depending on the context [8]. The service typically includes harm reduction education, overdose management, and referral to health and social services, including drug and addiction counselling and treatment [9]. Currently, there are over 90 SISs in operation around the world, with the majority located in Europe [10].

Most of the research evidence on the impacts of SISs available in the literature comes from just two facilities: Insite in Vancouver and the Medically supervised Injecting Centre (MSIC) in Sydney, Australia [8]. The available research demonstrates that SIS use can lead to decreases in high-risk injection practices, such as sharing injection equipment, rushed injecting, injecting in public, unsafe syringe disposal, and lack of alcohol swabbing of injection sites [8]. Other reported outcomes include low rates of overdose within SIS facilities and falling overdose rates in surrounding neighbourhoods [11–13], increased timely access to care for injection-related injuries and addiction treatment services [6, 7], and a lack of negative impacts on public order and drug-related crime [14, 15].

Despite the evidence that SISs can contribute to the health, safety, and engagement in care of PWID, there have been no new government-sanctioned sites established in Canada since Insite opened its doors in Vancouver in 2003. This lack of progress in establishing new sites does not reflect a lack of need, as drug-related harms remain prevalent in many urban centres across the country. HIV prevalence among Ottawa’s 3500 to 6000 PWID is estimated to be among the highest of any major Canadian city, at between 11 and 20 % [16]. HCV prevalence is estimated at 60 %, and as many as 98 % of PWID infected with HIV in Ottawa are co-infected with HCV [17]. The 2012 Toronto and Ottawa Supervised Consumption Assessment (TOSCA) study reported that 20 % of people who use drugs in Ottawa had experienced a non-fatal overdose in the previous 6 months and 14 % had injected with used needles [18]. In reviewing the evidence of local need, the TOSCA study recommended that Ottawa would benefit from the establishment of two SISs.

As the potential benefits of establishing an SIS in Ottawa are being increasingly recognized, issues of service access by PWID and context-sensitive service design must be explored. Previous Ottawa-based SIS feasibility studies indicated that 75–78 % of PWID in the city would be willing to use an SIS [1, 18, 19]. The most recent data on willingness to use an SIS in Ottawa was collected in 2005 highlighting the need for an up-to-date consultation with the communities of people who use drugs to inform new service development. There is also a need to describe the health risks currently faced by potential SIS users to inform the development of health and harm reduction services that meet current community needs.

We apply Rhodes’ ‘risk environment framework’ to inform an investigation of the risks for drug-related harms currently faced by potential users of an SIS in Ottawa [20]. In the Rhodes model, harm is produced through the interactions between individuals and the physical, social, economic, and policy environments in which they live. Both harm and harm reduction are considered to be contingent on these environmental contexts. This framework does not seek to guide a comprehensive investigation into all of the complex, dynamic, and multilevel aspects of the risk environment but rather to highlight the importance of environmental characteristics in shaping risks for drug-related harm [21, 22]. We undertook a survey to assess the risk behaviours and specific features of the risk environments currently faced by potential SIS users that are relevant to the need for an SIS in Ottawa and to service design. A deeper understanding of the current risk environments faced by PWID can also inform service development across the spectrum of harm reduction and healthcare provision. By developing a research cohort of PWID in the city, we seek to establish an evaluative baseline from which to monitor the impact of new health and harm reduction services in Ottawa, including SISs.

Urban communities that face issues of health inequity often develop an expert understanding of the complex risk environments that shape and interact with their daily lived experience to produce or minimize harm [23, 24]. Community-based participatory research (CBPR) combines the strengths of academic systems of inquiry with novel mechanisms of meaningful community participation and control at every stage of the research process to capitalize on this expert knowledge. CBPR also engages in an iterative cycle of research and action for social change [25]. This approach benefits research in a number of ways, including enhanced cultural sensitivity and validity of data collection and analysis, improved community trust and engagement, and contextually relevant health interventions [26]. Community engagement around risk environment knowledge generation has an additional benefit of effecting change within the social environment through community mobilization, relationship building, capacity building, and harm reduction advocacy. An enabling social environment can influence the success of new harm reduction services. We merge CBPR principles with quantitative cohort methods to describe the risk environments faced by PWID in Ottawa with the aim of informing new health and harm reduction service development, including SISs. This novel approach to community-based quantitative research can serve as a model for future harm reduction research that seeks to understand and simultaneously influence the risk environments faced by PWID.

## Methods

### Study design

The Participatory Research in Ottawa: Understanding Drugs (PROUD) study is a prospective cohort study that seeks to better understand the HIV risk environment among people who use drugs in Ottawa, Ontario. PROUD incorporates CBPR principles to actively engage a community advisory committee (CAC) consisting of people with drug use experience and their allies. Following a community information session hosted in collaboration with the Drug Users Advocacy League in May 2012, eight people with lived experience with drug use, three allied frontline support workers, and three ex officio representatives from organizations working to improve the health and rights of Ottawa’s drug-using communities were recruited into the CAC and began meeting monthly to develop and implement the study. CAC members receive an honorarium of $25 per meeting to support their contribution to all phases of the research process, including the research design, data collection, analysis, and knowledge translation stages.

Medical student volunteers were also engaged to assist with the recruitment phase of the study and to provide participants with a choice of interviewer. In total, 11 peers and 15 medical student volunteers received training in CBPR methods, HIV and harm reduction, interviewing skills, research ethics, and HIV point-of-care (POC) testing to prepare for active roles in the participant recruitment and data collection phases. The POC HIV test (bioLytical INSTI test) is a rapid test for the detection of HIV antibodies in fingerstick blood. Details of the training, survey development, and overall CBPR process in the PROUD study have been described previously [27].

### Participants

From March to December 2013, 858 people were enrolled into the PROUD study. Participants were eligible if they were aged 16 years or older, reported using injection drugs or smoking crack cocaine in the previous 12 months, and had been living in Ottawa for at least 3 months at the time of their interview. Of these participants, 593 were recruited in the ByWard Market area of Ottawa, which has the highest concentration of public syringe discards in the city [28] and is a likely site of a future SIS. The current analysis focuses on the 270 participants recruited in the ByWard Market who reported injecting drugs in the 12 months previous to their interview. A targeted, street-based recruitment strategy was developed by the CAC to capitalize on its expert knowledge of the often-hidden social networks of PWID in Ottawa. Recruiters wearing identifying lanyards approached potential participants in a variety of street-based and public locations, presented study information cards, followed a verbal recruitment script to determine interest and eligibility, and scheduled interview times.

### Data collection

At the research site located in the downtown core, trained peer or medical student researchers administered a one-time, tablet-based quantitative survey and a voluntary POC HIV test for those participants who did not self-report as HIV positive. English and French-speaking peer and medical student interviewers were available to provide participants with the choice of conducting interviews in either official language. Consent was obtained separately for participation in the survey, the POC HIV test, and for prospective follow-up through data linkages to provincial health service records available through the Institute for Clinical Evaluative Sciences (ICES). A voluntary opt-in approach was used to solicit participation in the POC HIV test and the data linkages. Participants received a cash honorarium of CAN$20.00 after completing the survey portion of the study. Ethical approval for this study was obtained from the Ottawa Hospital and the Ottawa Public Health Research Ethics Boards.

In the study development phase, the CAC developed survey themes and questions that reflected the experiences and priorities of their communities. These were then adapted to a survey format by the research coordinators and integrated into a risk environment framework. The survey questionnaire includes individual health, sociodemographic, and behavioural variables; aspects of the physical environment, including the locations of drug use; aspects of the social environment, including access to social services and peer groups and local policing practices such as redzoning and stop-and-search; aspects of the economic environment, including income, employment, and housing and homelessness; and aspects of the policy environment, including access to specific health, harm reduction, and social services [24]. The current descriptive analysis focuses on items in the questionnaire that characterize the risk environment of PWID and illustrate the need for an SIS in Ottawa including the following: types and frequencies of drugs injected, syringe-sharing practices, public injecting, requiring assistance to inject, lack of access to services, experience with overdose, and HIV and HCV infection status. The main outcome variable, willingness to use an SIS in Ottawa, is based on the question ‘Would you likely use a supervised injection site if one was opened in Ottawa?’. All participants were provided with a brief definition of an SIS before being asked about their willingness to use one.

### Statistical analysis

All analyses were conducted using SPSS Version 22.0. We undertook descriptive and univariate analysis to estimate the potential use of an SIS by PWID in Ottawa and to explore characteristics of the risk environments faced by those who are willing and not willing to use the service. To explore whether there is a statistical difference between the risk environments faced by those who are and those who are not willing to use an SIS, categorical and explanatory variables were analysed using the Pearson *χ*^2^ test, and continuous variables were analysed using the Wilcoxon rank sum test. Unadjusted odds ratios (ORs) with corresponding 95 % confidence intervals (CIs) were calculated and all reported *p* values are two-sided. Several variables included in the analysis have missing data due to participants refusing to respond. This is not surprising given the highly sensitive nature of the questionnaire. All refuse-to-answer responses were treated as missing data, rather than re-categorizing missing responses for analysis.

## Results

### Demographics, drug use, and health

Of the 593 participants recruited in the ByWard Market, 45.5 % (270) reported injecting drugs in the previous 12 months. Table 1 shows the demographic, drug use, health, and risk environment characteristics of these 270 participants. The median age was 42 years. Among these PWID, 15.2 % (41) spoke French as their first language and 49.3 % (133) lived in the ByWard Market or the larger Lowertown area of Ottawa. Almost one quarter of PWID participants were female (62 or 23.0 %), and nearly one fifth identified as Aboriginal (47 or 17.4 %). When asked their opinion on SISs, 84.8 % (229) thought there should be an SIS in Ottawa, and 75.2 % (203) reported that they would use an SIS if one were to open in the city. More than half of the 203 potential SIS users reported that they would use the service daily if it opened right away (103 or 50.7 %). Among those participants who expressed willingness to use an SIS (*n* = 203), 72.9 % (148) injected morphine, 70.4 % (143) injected cocaine, 56.2 % (114) injected crack-cocaine, and 45.8 % (93) injected heroin within the previous year. Almost half (46.3 %) reported injecting opiates a few times a week or more. When questioned about health outcomes, 74.9 % (152) of potential SIS users reported ever having been diagnosed with a mental health illness, 63.5 % (129) tested positive for HCV at their last test, and 12.8 % (26) self-reported that they were HIV positive.

**Table 1:** Demographic, behavioural, and risk environment characteristics disaggregated by willingness to use an SIS in Ottawa^a^

	Intention to use an SIS in Ottawa; no. (and %) of participants^b^	
Characteristic^c^	Yes	No
	*n* = 205	*n* = 67
Demographics, drug use, health		
Age (median)	42.0	44.0
Female	52 (25.4)	10 (14.9)
French-speaking	30 (14.6)	11 (16.4)
Aboriginal identity	39 (19.0)	8 (11.9)
Market/Lowertown residence	99 (48.3)	35 (52.2)
LGBTQ identity	29 (14.1)	2 (3.0)
Injected crack cocaine	114 (55.6)	17 (25.4)
Injected speedball	63 (30.7)	8 (11.9)
Injected fentanyl	79 (38.5)	11 (16.4)
Injected heroin	93 (45.4)	15 (22.4)
Injected dilaudid	133 (64.9)	29 (43.3)
Injected morphine	148 (72.2)	36 (53.7)
Injected cocaine	143 (69.8)	35 (52.2)
Injected opiates a few times a week or more	94 (45.9)	20 (29.9)
Overdosed	66 (32.2)	13 (19.4)
Ever tested HIV positive	26 (12.7)	6 (9.0)
Last hep C test positive	129 (62.9)	32 (47.8)
Mental health diagnosis (ever)	152 (74.1)	52 (77.6)
Attempted suicide	17 (8.3)	4 (6.0)
Would use SIS daily	103 (50.2)	NA
Physical risk environment		
Injects in public	81 (39.5)	17 (25.4)
Used unknown needle	34 (16.6)	7 (10.4)
Injected with used needle	50 (24.4)	10 (14.9)
Injects with other people	176 (85.9)	44 (65.7)
Assistance to inject	81 (39.5)	18 (26.9)
Social risk environment		
Ever redzoned^d^	102 (49.8)	20 (29.9)
Stopped/searched by police	151 (73.7)	45 (67.2)
Kept overnight or longer in jail	97 (47.3)	32 (47.8)
Policy risk environment		
Trouble accessing new needles	39 (19.0)	6 (9.0)
Access addiction treatment	80 (39.0)	26 (38.8)
Have regular doctor	113 (55.1)	33 (49.3)
Sought care in hospital/ER	89 (43.4)	27 (40.3)
Tested for HIV (ever)	188 (91.7)	57 (85.1)
Economic risk environment		
Received drugs, $, gifts for sex	38 (18.5)	6 (9.0)
Sex work primary source of income	13 (6.3)	4 (6.0)
Unstably housed	123 (60.0)	32 (15.6)
Homeless past 12 months	164 (80.0)	47 (22.9)

^a^Percentages are calculated within the subgroups of participants willing or not willing to use an SIS

^b^Because of missing responses, the data for some characteristics do not sum to 272

^c^All variables are reported for the previous 12 months unless otherwise specified

^d^Redzones are defined as any geographical area or neighborhood where law enforcement had restricted the participant’s movement

### Risk environment characteristics

Public injecting is a key marker of the physical risk environment and was common among potential SIS users at 39.5 % (81) [24]. Interactions with local policing practices, a characteristic of the social risk environment, were also common with 74.4 % (151) having been stopped and searched by the police without arrest and 47.8 % (97) having been kept overnight or longer in prison, jail, or a detention centre in the previous 12 months. Aspects of the policy risk environment include availability and access to health and harm reduction services. Among potential SIS users, 19.2 % (39) had trouble accessing new needles, 39.4 % (80) had accessed addictions treatment in the past 12 months, and 43.8 % (89) had sought care in a hospital/ER in the previous year. Characteristics of the economic risk environment include housing, employment, and income. As many as 94.1 % (191) of potential SIS users had ever been homeless, and 60.6 % (123) were unstably housed at the time of their interview (defined as living in a rooming house, shelter, or on the street/homeless). Almost half had a monthly income of $999 or less (48.8 %), 49.7 % (101) relied on a food bank or shelter for 75 % or more of their meals, and 57.1 % (116) had not completed high school.

### Univariate analysis

In univariate analysis, those willing to use an SIS were more often younger (42 vs. 44 years, *p* = 0.04), identified as LGBTQ (OR = 5.61, 95 % CI = 1.30–24.19), injected in public (OR = 1.98, 95 % CI = 1.06–3.70), injected with other people (OR = 3.23, 95 % CI = 1.66–6.27), required assistance to inject (OR = 1.84, 95 % CI = 1.00–3.38), had overdosed in the past 12 months (OR = 2.00, 95 % CI = 1.02–3.92), and had tested positive for HCV at their last test (OR = 2.13, 95 % CI = 1.16–3.91) (Table 2).

## Discussion

Potential uptake of an SIS in Ottawa is high with more than three quarters of PWID PROUD study participants reporting a willingness to use the service. This is consistent with previous feasibility studies which indicate a relatively stable demand for the service over time [1, 18, 19]. A study on the validity of such feasibility assessments in Vancouver found that reporting an initial willingness to use an SIS was a significant predictor of later use of the service, with 72 % of those who reported an initial willingness later attending the programme [29]. The sub-population of PWID who are likely to use an SIS currently faces complex risk environments for drug-related harm. Many of these harms and several aspects of the risk environment could potentially be addressed through the development of supervised injection services that are responsive to the local context.

**Table 2:** Risk environment comparison by willingness to use a supervised injection service in Ottawa^a^

	Intention to use an SIS in Ottawa; no. (and %) of participant^b^		
Characteristic^c^	Yes	No	OR (and 95 %CI)^d^
	*n* = 205	*n* = 67	
Age	42.0	44.0	*p* value = 0.04
Sexual identity			
LGBTQ	29 (93.5)	2 (6.5)	5.61 (1.30–24.19)
Straight	168 (72.1)	65 (27.9)	
Injects with other people			
Yes	176 (80.0)	44 (20.0)	3.23 (1.66–6.27)
No	26 (55.3)	21 (44.7)	
Injects in public			
Yes	81 (82.7)	17 (17.3)	1.98 (1.06–3.70)
No	113 (70.6)	47 (29.4)	
Assistance to inject			
Yes	81 (81.8)	18 (18.2)	1.84 (1.00–3.38)
No	120 (71.0)	49 (29.0)	
Last hep C test			
Positive	129 (80.1)	32 (19.9)	2.13 (1.16–3.91)
Negative	51 (65.4)	27 (34.6)	
Overdosed			
Yes	66 (83.5)	13 (16.5)	2.00 (1.02–3.92)
No	137 (71.7)	54 (28.3)	

*OR* odds ratio, *CI* confidence interval

^a^Percentages are calculated on the basis of the sum across each row

^b^Except where indicated otherwise. Because of missing responses, the data for some characteristics do not sum to 272

^c^Variables are reported for the previous 12 months unless otherwise specified

^d^For each categorical variable, the reference category is the second category

The demographic, health, and drug use variables described here have implications for supervised injection service development. Those PWID who were younger or identified as LGBTQ expressed willingness to use an SIS more frequently than older individuals and those who did not identify as LGBTQ. This illustrates the potential of an Ottawa-based SIS to reach and engage communities who face multiple and intersecting oppressions simultaneously. These findings may indicate an increased awareness and support for particular harm reduction services among these groups, though the reasons for such high interest in using an SIS remain to be explored.

In the year preceding their interview, nearly a quarter of potential SIS users in Ottawa had used a syringe that they knew had been used by someone else and nearly half injected opiates several times a week or more. Frequent injecting and syringe sharing are associated with increased risk of transmission of blood-borne infections [30]. SIS use in other contexts has been associated with a decrease in syringe sharing [31]. There is also evidence of significant polysubstance use in the cohort. Polysubstance use has been associated with negative health outcomes including HCV infection and overdose [32–34]. Efforts to engage and accommodate frequent injectors and polysubstance users in future SIS and other harm reduction services in Ottawa should be prioritized.

Potential SIS users also experienced a high burden of negative health outcomes, with high rates of mental health diagnoses (74.9 %) and HIV infection (12.8 %) and a higher burden of overdoses and HCV infection than those unwilling to use the service. Insite has had a measurable impact on the burden of overdoses in Vancouver, demonstrating a 35 % reduction in the fatal overdose rate surrounding the site just 2 years after opening [11]. The TOSCA study estimates that 6–10 new HIV infections and 20–35 new hepatitis C infections could be averted per facility per year by the first two SIS facilities in Ottawa, although the prevention potential of additional facilities is considerably lower [18]. A recent analysis of the costs and benefits that could be expected if Ottawa were to open an SIS found that the service appears to be an efficient and effective use of financial resources based on savings from the prevention of HIV and HCV cases alone [35]. However, the potential of an SIS to improve health outcomes is limited by the suitability of the service model to the needs of potential users. In Vancouver’s SIS, nurses and other clients are not permitted to physically assist a client with an injection. This regulation has been associated with reduced access to the site among some high-risk groups [36]. In the current study, potential SIS users reported requiring assistance to inject more frequently than PWID who did not report a willingness to use the site. An SIS in Ottawa should consider developing approaches and regulations for engaging and supporting PWID who require assistance to inject.

When compared to participants who did not report willingness to use an SIS, potential SIS users injected in public more frequently. Public injection locations are often characterized by unhygienic conditions and exposure to street violence and the police, which can lead to rushed injecting [37]. Because of these characteristics of the physical environment, public injecting has been associated with negative health outcomes including overdose and HIV and HCV infection [38]. In the 12 weeks following the opening of Vancouver’s SIS, there was a significant reduction in the number of drug users injecting in public, publicly discarded syringes, and injection-related litter [39]. An SIS in Ottawa could provide a safer physical environment for many PWID currently exposed to public-injection-related risks and improve public order in the surrounding neighbourhood [39]. Limitations to accessing the service, such as long wait times, were independently associated with persistent public injection in a cohort of IDU recruited from the SIS in Vancouver, demonstrating the importance of accessibility in harnessing the potential of SISs to alter the physical environments of risk for PWID [40].

Aspects of the social environment examined in this study include experiences of policing practices. Three quarters of potential SIS users had been stopped and searched by the police in the previous 12 months, and half had been redzoned, meaning their movement in a particular geographical area or neighbourhood had been restricted by law enforcement. If these policing practices continue in the area where a new SIS is opened, they could have important negative implications for service access [20]. The police are therefore key stakeholders that should be engaged in developing an enabling social environment for harm reduction.

Other important aspects of the social environment are the knowledge and relationships within the communities of PWID which shape community norms and support effective mobilization for harm reduction advocacy [24]. The application of CBPR principles to the PROUD research process helped to support the development of new community leadership and increased mobilization on harm reduction issues, expanding the reach of existing peer advocacy efforts and supporting the development of new relationships of solidarity. These are described by Duff as key elements of an ‘enabling’ social environment for harm reduction [41]. CAC members participated in significant community outreach and education throughout the study recruitment and knowledge translation. Study participants responded positively to seeing community members in leadership and research roles and generally did not state a preference for medical student or community member interviewers or HIV testers. The broader community impacts of this model have been visible in the high levels of PWID attendance at thematic community forums and advocacy events where ten plain-language PROUD newsletters have been released since October 2012. At these forums and advocacy events, PROUD study results have been presented by the CAC to community members and service providers for feedback and discussion. The link between research and social action that is forged through CBPR methods can reduce harm in its own right and help foster a social environment conducive to effective harm reduction [41].

Aspects of the economic risk environment, including homelessness, food insecurity, and lack of access to education, were also highly prevalent among potential SIS users. SISs in other contexts have had success in engaging PWID who face these complex social determinants of health [8]. In a review of 14 studies from Vancouver, Sydney, Geneva, Madrid, and Barcelona, the majority of the most frequent SIS users had a previous history of incarceration and faced issues of housing insecurity and unemployment [8]. In terms of the policy environment, access to healthcare was not found to be significantly different between those who would and those who would not use an SIS, though both groups reported barriers to care. Nearly one fifth of potential SIS users had trouble accessing new needles, and more than 60 % had not accessed any addiction treatment in the past year. The high rate of hospital emergency room use (43.9 %) has been observed among other PWID populations and is linked to barriers to accessing primary care services [2, 42]. Use of an SIS has been shown to increase timely access to care for injection-related injuries and infections [6] and increase uptake of addiction treatment services [7]. Economic risks and other barriers to accessing care can interfere with access to harm reduction services, including SISs. Ongoing engagement of PWID in harm reduction research and service design can help to identify and address context-specific access barriers as they arise.

Although an SIS can improve engagement across the spectrum of health and harm reduction services through service referrals and integration, it is clear that not all PWID would be willing to use an SIS and not all who are willing would have access. Though the focus of this paper is on the risk environments faced by potential users of SIS services, it is important to consider the potential unmet needs of those who report a lack of willingness to use the service. In the current study, those not willing to use an SIS if one were available more frequently reported not injecting in public and not injecting with other people. A previous study of willingness to use SIS services in Vancouver identified injection at home, already having a safe place, and willingness to inject in private as the major reasons not to use the SIS [43]. The harm reduction needs of those who inject alone in private residences are important to explore in future research. Other context-specific barriers and reasons for not accessing SIS services should also be investigated.

There are several limitations to the current study. Though probability sampling is not feasible in this hard-to-reach population, our street-based and peer-driven recruitment approach has the potential to identify populations with the highest health risk behaviours [44]. The CAC elected to recruit during daylight hours due to group safety concerns, which could reduce the representation of sex workers and other community members who are primarily available at night. Recruiters targeted street-involved PWID, which mirrors the target population of an SIS. However, this targeting leads to an overrepresentation of homeless and socially marginalized PWID, which may not be representative of all PWID in Ottawa or in other settings. An additional limitation is our outcome measure, which assesses hypothetical use of a future site. To further explore the uptake and impact of an SIS on drug-related harm in Ottawa, service providers should develop and implement a rigorous monitoring and evaluation programme to assess the real benefit of a site once opened.

As the survey relies on self-reported data, study results may be influenced by social desirability bias. Since respondents were asked about practices that are highly stigmatized or illegal, this bias would likely result in an underestimation of high-risk practices. Missing data is generally the result of participant non-response to questions they would prefer not to answer and would also likely result in an underestimation of high-risk practices.

The current study reports descriptive and univariate statistics to give a preliminary overview of some features of the risk environments faced by PWID in Ottawa to inform new health and harm reduction service development. However, this descriptive approach precludes the identification of independent associations between various risk factors and their influence on drug-related harms and harm reduction service access. The use of cross-sectional data limits our ability to predict willingness to use an SIS based on specific health risks or service user characteristics. Rhodes [24] highlights the difficulty of isolating the individual effects of risk environment characteristics on harm and access to harm reduction services given the non-linear relationships that exist between multilevel characteristics of these environments, which interact with each other and with individual agency to cause dynamic changes over time and place. Future research should seek to characterize these dynamic associations between different aspects of the risk environment and the use and effects of harm reduction services.

Since this study captures self-report of different aspects of the risk environment as experienced by individuals, it examines the ‘micro’-level of Rhodes’ [20] environmental influence categorization, omitting macro-level elements of the risk environment such as national and regional policies, laws, and economic resources. As a result, this study captures only a fraction of the complex contextual elements that shape or interact with risk for harm and access to harm reduction among PWID. The CBPR methods used in this study ensure that the aspects of the risk environment examined reflect the priorities of the communities of PWID who would be affected by a future SIS.

## Conclusion

Like many cities across Canada, PWID in Ottawa continue to face significant risks of drug-related harm and barriers to accessing appropriate care. This study demonstrates that there would be high uptake of an SIS if established in Ottawa, that the service has the potential to reach its target population of marginalized PWID, and that an SIS tailored to the context-specific needs of the local population could potentially address important aspects of the risk environments faced by PWID. This study has implications for harm reduction beyond the Ottawa context in two respects. First, it highlights the utility of the risk environment framework for informing quantitative cohort study research investigating the context-specific harm reduction needs of PWID. Second, it demonstrates the potential to merge quantitative survey methods with CBPR principles to foster meaningful community ownership and control over the research process and support communities of PWID in identifying and advocating for their own service needs.
